# Development and validation of a curriculum for laparoscopic supracervical hysterectomy

**Published:** 2020-08-05

**Authors:** JM Goderstad, E Fosse, L Sandvik, M Lieng

**Affiliations:** Department of Surgery, Sørlandet Hospital, Sykehusveien 1, 4838 Arendal, Norway;; The Intervention Centre, Oslo University Hospital, 0424 Oslo, Norway;; Institute of Clinical Medicine, University of Oslo, 0316 Oslo, Norway;; Center for biostatistics and epidemiology, Oslo University Hospital, 0373 Oslo, Norway;; Division of Obstetrics and Gynecology, Oslo University Hospital, 0424 Oslo, Norway.

**Keywords:** Laparoscopy, simulation training, proficiency-based training, certification, curriculum

## Abstract

**Study Objective:**

To develop and validate a three-step curriculum for laparoscopic supracervical hysterectomy (LSH) designed for a busy clinical setting.

**Nethods:**

Single-centre, prospective, cohort study. Twelve eligible gynaecological trainees were included (group 1). The theoretical part (step 1) was a validated multiple-choice test. The practical part (step 2) consisted of five tasks on a virtual reality simulator. The participants had to reach a pre-defined proficiency level before advancing to performing a LSH (step 3). The validation of the curriculum was based on the surgical performance. The surgical procedure was recorded and assessed by two experts using Global Operative Assessment of Laparoscopic Skills (GOALS) and Competence Assessment Tool – Laparoscopic Supracervical Hysterectomy (CAT-LSH). The scores were compared with scores from gynaecological trainees who performed their first LSH without virtual reality simulator training (group 2).

**Results:**

Ten trainees completed the curriculum and performed a LSH that was recorded and evaluated. Mean duration of the training period (step 1 and 2) was 57 days (SD 26.0), and mean training time spent on the simulator to reach the pre-set proficiency level was 173 min (SD 49). The mean GOALS score was 18.5 (SD 5.8) in group 1 and 13.6 (SD 3.3) in group 2, p=0.027. The mean CAT-LSH score of the performance of the hysterectomy was 42.1 (SD 6.9) in group 1 and 34.8 (SD 4.3) in group 2, p= 0.009.

**Conclusions:**

Trainees who completed the curriculum appeared to have a higher performance score compared with trainees who did not perform structured training.

## Introduction

It is recommended that surgical residents undergo initial laparoscopic skills training outside the operating room (Reznick et al., 2006; Seymour et al., 2002; Larsen et al., 2009; Campo et al., 2014; Gala et al., 2013; Sroka et al., 2010). However, in spite of convincing research reports demonstrating the advantages of structured laparoscopic skills training, the implementation of structured training curricula remains challenging. This might be explained by limiting factors such as logistics, equipment, clinical tasks and working hours.

Along with the increased implementation of minimally invasive surgery for common surgical procedures, there has been a concomitant reduction of participation of junior-level residents. The trend of less surgical procedures among residents has significant implications for surgical resident education (Mullen et al., 2016). It is consequently essential that a part of the surgical training takes part outside the operating theatre. Otherwise, the practical skills for future surgeons might be negatively influenced.

For a surgical training curriculum to be successful, several elements are required. It is essential that the curriculum contains a cognitive component, a practical component, and subsequent supervised training in the actual clinical setting (Strandbygaard et al., 2014). A successful surgical training curriculum also depends on trainee, faculty and employer commitment (Stefanidis et al., 2009). Scoring systems and proficiency-based training can give summative and formative feedback that motivates the trainees. Logistics that facilitates distributed training with defined training hours is essential. Hence, the need for structured training in a busy clinical setting must be acknowledged by the employer in order to implement a structured training programme successfully. Education of health personnel, including registrars, is one of the major tasks of teaching hospitals. However, in our experience, structured training for registrars is often not prioritised in clinical departments. The objective of the study was to develop and validate a curriculum for laparoscopic supracervical hysterectomy (LSH) that increased the trainees’ surgical performance and was designed to be feasible to completed in a busy clinical setting.

## Methods

### 

**Figure 1:**
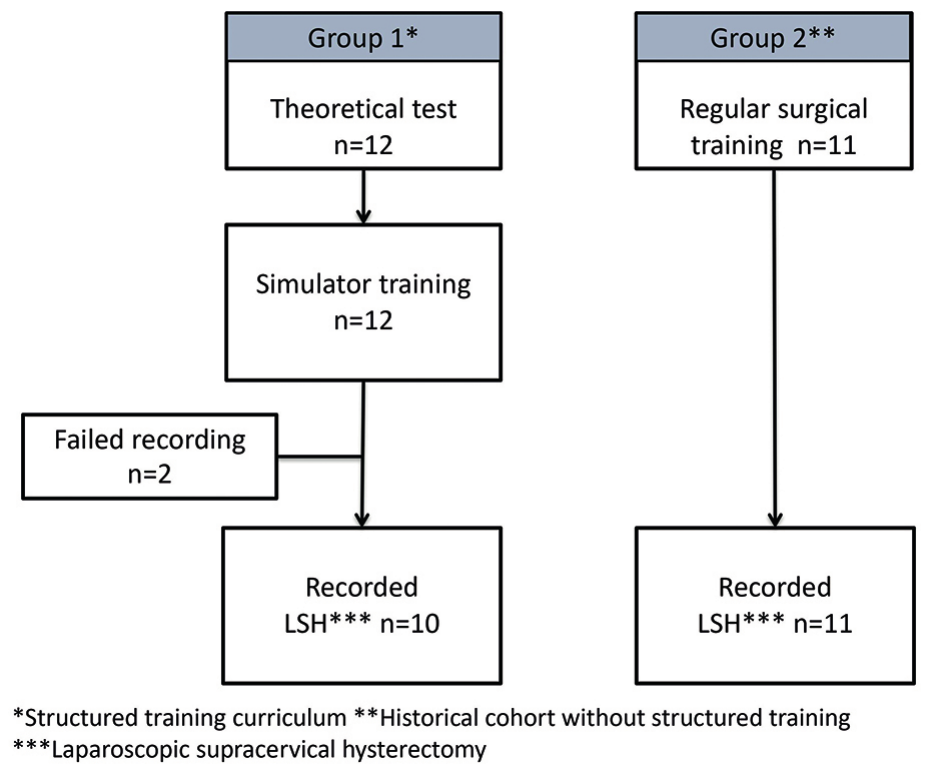
— Study flow chart.

The study was a single-centre, cohort study performed at a Norwegian university hospital. All junior trainees at the department of gynaecology were invited to participate. To be eligible for study participation, they had to be able to perform basic laparoscopic procedures supervised by a consultant, and about to move on to perform more complex procedures like hysterectomy. Registrars eligible for study inclusion should not have performed more than 50 laparoscopic procedures previously, and they should plan to continue employment at the department for the next six months. Prior to inclusion, all study participants received written information about the study, and they signed an informed consent for participation. The study participants were followed up according to the study flowchart (Figure 1).

The curriculum consisted of three steps. All study participants first underwent a theoretical knowledge multiple-choice test within basic laparoscopy (step 1). We used questions from a test previous published by Strandbygaard et al., 2013. As the curriculum was designed for laparoscopic supracervical hysterectomy (LSH), we added six questions related to this particular procedure. These questions were evaluated by three international experts in gynaecological laparoscopy before they were included in the theoretical test. The aim of the theoretical test was to stimulate the study participants to obtain theoretical knowledge related to laparoscopy in general, as well as the specific procedure before they started the training and performed the first surgical procedure. Wrong answers in the test did not have any consequences for the registrars, but all wrong answers were discussed to make the registrar able to give the correct answer.

The study participants then underwent a structured individual laparoscopic training programme (step 2). The training was carried out using the Simbionix, LAPmentor Express, 3D, VR simulator. At the first training session, all study participants were given a standardised hands-on introduction to the Simbionix LAPmentor system, and an oral presentation as well as a video presentation of the different tasks included in the training programme. The tasks had varying complexity and consisted of three basic skill tasks, a salpingectomy and a LSH. We aimed for distributed training, meaning short training periods, with rest periods in between. We planned three repetitions of each task at each training session and estimated that the participants needed at least three sessions to reach the pre-set proficiency level.

### Description of the tasks included in step 2:

#### Task 1: Two-handed manoeuver

**Figure 2:**
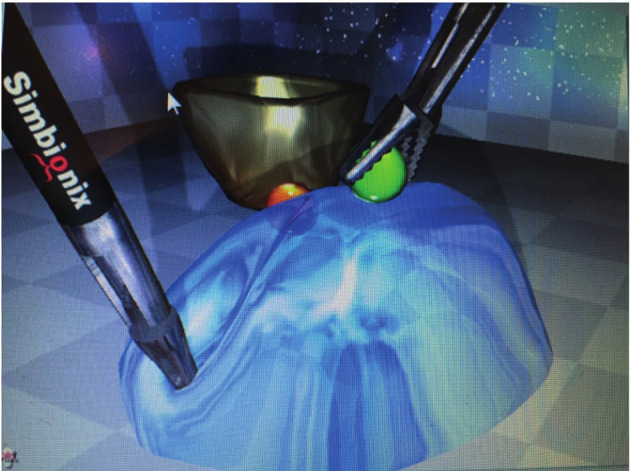
— Task 1. Two-handed manoeuver.

The task included exposure of nine balls embedded in jelly. A correctly exposed ball changed the colour from red to green. All balls then had to be grabbed and placed into a basket (Figure 2).

This is a coordination task involving speed and precision. The objectives are to improve advanced bimanual skills, practice instrument manipulation and eye-hand coordination, and acquire tissue- handling skills.

The parameters measured were time (s), number of balls in the basket (n), total path length (cm) and instrument movement (number). In addition, number of errors was registered (only green balls should be grabbed).

#### Task 2: Peg transfer

**Figure 3:**
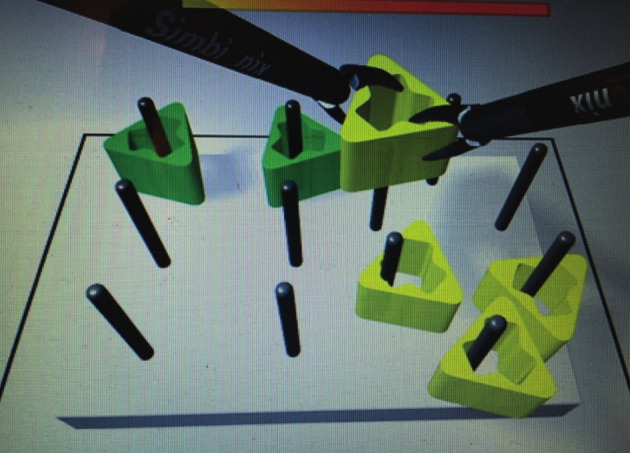
— Task 2. Peg transfer.

The participants lifted six objects from a pegboard with the left hand, transferred the object to the right hand, and placed them over the pegs on the pegboard. The process was then reversed (Figure 3).

The objectives are improved eye-hand coordination, use of both hands and depth perception, and the measured parameters were total time (s) and number of successfully moved objects (without loss and correctly placed on the pegboard) (n).

#### Task 3: Pattern cutting

The participants used a grasper to apply traction exposing the best angle for the dominant hand to cut in the marked circle with accuracy.

The objective of this task is use of both hands and accuracy. The parameters measured were total time (s) and errors (any deviation from the drawn line).

#### Task 4: Left side salpingectomy

The participants used a grasper, scissors, and a bipolar forceps to remove the left tube. The total time used on the task (min) was registered. In case of an error (bleeding), it had to be corrected before commencing the salpingectomy.

#### Task 5: Modified LSH

**Figure 4:**
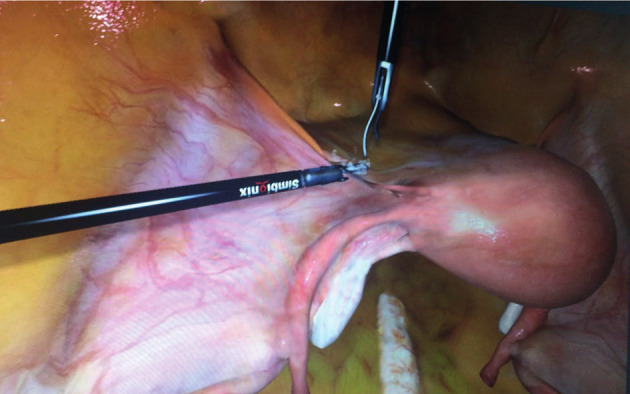
— Task 5. Laparoscopic supracervical hysterectomy.

The participants were introduced to a step-by-step strategy of the procedure starting on the left side and including: identification and division of the round ligament, identification of the anterior leaf of the broad ligament and progressive cauterisation of the ligament towards the middle medially paying attention to the bladder, coagulation and division of the proper ovarian ligament and the fallopian tube, division of the posterior leaf of the broad ligament and identification, coagulation, and division of the uterine vessels (Figure 4). The same steps were then performed at the right side. Finally, the cervix was exposed and the participant marked the correct level for amputation. In this task, total procedural time (min), total path length (cm), instrument movements (n) and errors (bleeding and improper respect of tissue/tissue handling) were registered. The registration started when the participant took hold of the round ligament on the left side.

### 

The registered parameters in each task were combined, giving a total score of performance for evaluation of skills of each study participant in each task.

The study participants performed the tasks independently. An instructor (JMG) was present during all training sessions in order to give feedback, assist the study participants in case they needed guidance on the simulator system, or information regarding the different tasks.

The total training period (step 2) was aimed to last between two and six weeks. The different tasks were repeated until the participant reached the pre- set level of proficiency. This level was defined by results from a previous study including experienced laparoscopic surgeons performing the same tasks (Goderstad et al., 2019).

When the study participants reached the required proficiency level, they performed a LSH assisted by an experienced surgeon (step 3). The procedure was recorded and evaluated by two blinded experienced surgeons, using two validated scoring systems, Global Operative Assessment of Laparoscopic Skills (GOALS) and Competence Assessment Tool - Laparoscopic Supracervical Hysterectomy (CAT- LSH) (Goderstad et al., 2016). In GOALS, the performance within six domains (depth perception, bimanual dexterity, efficiency, tissue handling, autonomy and level of difficulty) are evaluated and given a score from 1 – 5 (Table I). The scores of each domain are added to a total score. Hence, the lowest possible GOALS score is 6, and the highest 30.

**Table I:** — Global Operative Assessment of Laparoscopic Skills (GOALS).

Domains	1	2	3	4	5
Depth perception	Constantly overshooting target, hits backstop, wide swings, slow to correct.		Some overshooting or missing plane but corrects quickly.		Accurately directs instruments in correct plane to target.
Bimanual dexterity	Use of one hand, ignoring non-dominant hand, poor coordination between hands.		Use of both hands but does not optimize interactions between hands to facilitate conduct of operation.		Expertly uses both hands in a complementary manner to provide optimal working exposure.
Efficiency	Uncertain, much wasted effort, many tentative motions, constantly changing focus of operation, or persisting at a task without progress.		Slow, but planned and reasonably organized.		Confident, efficient and safe conduct of operation, maintaining focus on component of procedure until better done approach.
Tissue handling	Rough, tears tissue by excessive traction, injures adjacent structures, poor control of coagulation device, grasper frequently slips off.		Handles tissues reasonably well with some minor trauma to adjacent tissues, eg coagulation of non- target tissue, occasional slipping of grasper.		Handles tissue very well with appropriate traction on tissues and negligible injury of adjacent structures. Uses energy sources appropriately but not excessively.
Autonomy	Unable to complete entire procedure, even in a straight forward case and with extensive verbal guidance.		Able to complete operation safely with moderate prompting.		Able to complete operation independently without prompting.
Level of difficulty	Easy exploration and dissection.		Moderate difficulty (eg, mild inflammation, scarring, adhesions, obesity, severity of Disease.		Extremely difficult (eg, severe inflammation, scarring, adhesion, obesity, or severity of disease).

Evaluation using CAT-LSH includes the scoring of four procedure specific surgical variables within four different steps of the LSH procedure (Table II). All variables in each step are given a score from 1-4, and then added to a total score. The lowest possible CAT-LSH score is consequently 16, and the highest 64. The results were then compared with a cohort of trainees at similar surgical competence levels who performed their first LSH assisted by an experienced surgeon without preforming any preoperative training on a simulator (group 2) (Goderstad et al., 2016). The inclusion criteria for this group were identical as for the training group (group 1); the surgical procedure was performed in the same standardised manner and by using similar instruments, and the same two blinded experienced surgeons evaluated the recorded procedures using the two same validated scoring systems (GOALS and CAT-LSH).

**Table II:** — Competence Assessment Tool-Laparoscopic Supracervical Hysterectomy ( CAT-LSH) (_1/2_) .

Tasks	Instruments	Tissue	Errors	End-product
**Lig. mobilisation.** Adequate exposure and dividing of the round ligament and the broad ligament.	**Use of instruments:** **Table:** Uncoorninated Stiff and uncontrolled movements, overshooting Hesitant Controlled movements but hesitant and inefficient Skillfull Smooth, controlled and meaningfull movements Versatile Masterfull instrument use, effective movements n/a	**Use of non-dominant hand :** **Table:** Sagnant NDH does not move Lagging NDH is adjusting with delay or without efficency Meaningfull Meaningfull adjustment of NDH to improve exposure Forward looking Strategic and intelligent adjustment by NDH n/a	**This task was performed with:** **Table:** Complication Bleeding Near miss To close to the pelvic wall.Bloody dissection. No damage No damage Tissue protective Performed with best possible tissue protection n/a	**Was the ligaments divided safely?** **Table:** No Divided in an unfavorable distance from the uterus. Vaguely No access to the vesicouterine space Yes Safe divition Anatomically Crystal clear demonstration of anatomy n/a
**Adnexa** The proper ovarian ligament and fallopian tube are coagulated and divided	**Use of bipolar forceps, scissors or harmonic:** **Table:** Uncoordinatet Stiff and uncontrolled movements, overshooting Hesitant Controlled movements but hesitant and inefficient Skillfull Smooth, controlled and meaningfull movements Versatile Masterfull instrument use, effective movements n/a	**Use of non-dominant hand :** **Table:** Sagnant NDH does not move Lagging NDH is adjusting with delay or without efficency Meaningfull Adjustment of NDH to improve exposure Forward looking Strategic and adjustment by NDH n/a	**This task was performed with:** **Table:** Complication Bleeding Near miss To close to the ovarian tissue/uterus No damage No damage to the ovaries. Tissue protective Performed with best possible tissue protection. n/a	**Was the proper ovarian ligament and the fallopiantube coagulated and divided correctly?** **Table:** No Divided in an unfavorable distance from the uterus. Vaguely To close to the ovarian tissue. Yes Main structures identified and divided correctly. Anatomically Crystal clear demonstration of anatomy n/a
**Vascular controle** Identify the uterine vessels, coagulate and divide them	**Use of bipolar forceps and scissors:** **Table:** Hazardios Uncontrolled movements Laborious Awkward and repeated unnecessary attempts Efficent Instruments accurately placed and engaged Masterly Highly efficient and safe use of instruments n/a	**Dissection of the uterine vessels:** **Table:** Hazardios Insufficient view, uncontrolled movements Laborious Repeated unnecessary attempts Efficent Instruments accurately placed and engaged Masterly Highly efficient and safe use of instruments n/a	**This task was performed with:** **Table:** Complication Bleeding, the vessels was not identified Near miss Several attempts at several places to secure the vessels Efficent Visualisation of the vessels Ideal precision Smooth and efficient dissection n/a	**Are the vessels identified and secured at the right level ?** **Table:** Uncontrolled Vessels not secured Imprecise Vessels not accurately secured Safe Vessels secured before divition Flawless Perfectly secured before divition n/a

### Sample size calculation:

The sample size calculation was based on the total GOALS score. In a previous simulation study with eight inexperienced and 13 experienced surgeons, mean difference in GOALS between the groups was 5.8, and the standard deviation in each group was 4.0 (Larsen et al., 2012). We assumed that we would obtain equivalent GOALS scores in our study, and planned to use independent samples t-test with 5% significance level when comparing the groups. It may then be shown that in order to achieve 80 % test power, at least 10 trainees had to be included in each study group. Assuming a 20 % drop-out rate, we decided to include 12 trainees in the intervention group (group 1). Notably, we already had access to data from 11 inexperienced trainees (group 2) from a previous study (Goderstad et al., 2016).

### Statistical analyses:

The data was analyzed using SPSS 23.0 (SPSS Inc., Chicago, IL). All statistical tests were performed two sided at the significance level of 5 %. A two-sided Independent t-test was used when comparing normally distributed continuous variables.

### Ethical approval:

The study was conducted in accordance with the Declaration of Helsinki and national and local regulations. The Regional Committee for Medical Research Ethics in eastern and southern Norway and the personal data officer at Oslo University Hospital approved the study protocol.

## Results

**Figure 5:**
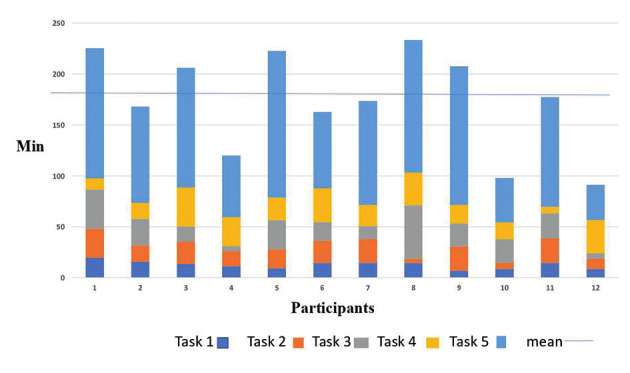
— Time spent to reach proficiency level for each task.

**Figure 6:**
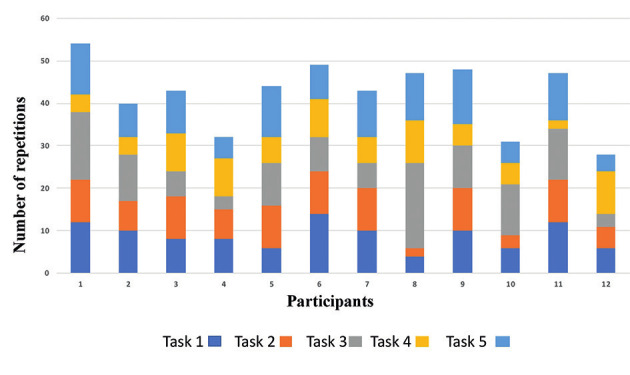
— Number of repetitions needed to reach proficiency level for each task.

The study took place from March 2015 to August 2016. All 12 study participants completed the theoretical test and the structured simulation training and reached the required proficiency level. During the surgical procedure (step 3), recording of the procedure failed in two cases, leaving 10 recorded procedures available for blinded evaluation (Figure 1). The mean duration of the training period (step 1-2) was 57.0 days, (SD 26.0). Mean time spent on the different tasks was 12.5 min (SD 3.7), 17.8 min (SD 7.7), 22.7 min (SD 13.5), 22.9 min (SD 10.1) and 97.8 min (SD 36.9) for task 1, 2, 3, 4 and 5, respectively. Mean total duration for all five tasks was 173.0 min (SD 49.0) (Figure 5). The average number of repetitions to reach proficiency level for all tasks was 42.0 (SD 8.0) Mean number of repetitions was 8.8 (SD 3.0), 7.8 (SD 3.0), 9.6 (SD 4.8), 6.5 (SD 2.7), and 9.1 (SD 3.0), for task 1, 2, 3, 4 and 5, respectively (Figure 6).

In the validation of the curriculum, the mean scores following the assessment by the two experts were 18.5 (SD 5.8) for GOALS, and 42.1 (SD 6.9) for CAT-LSH, respectively. The scores in the group of trainees who did not undergo the structured training (group 2) was 13.6 (SD 3.3) for GOALS and 34.8 (SD 4.3) for CAT-LSH.

The differences in GOALS and CAT-LSH mean scores between group 1 and 2 were statistically significant, p=0.027 for GOALS and p= 0.009 for CAT-LSH.

## Discussion

The training curriculum appeared to have a positive effect on the surgical performance, as trainees who completed the curriculum had a higher performance score on their first laparoscopic hysterectomy compared to trainees without structured training. Our results are supported by findings of previous studies, evaluating the surgical performance following structured training outside the operating theatre (Larsen et al., 2012). By using the mean of the experts’ performance it is possible to set a proficiency level for any training procedure on a box, a simulator or tasks in a dry or wet lab (Goderstad et al., 2019). Training in order to reach a defined proficiency level consequently appears to improve the surgical performance in the operating room. This finding is of importance to clinical practice. In 2014, six leading international societies within gynaecology recommended that each hospital teaching endoscopic surgery should make an endoscopic dry lab for training available in order to improve the proficiency of the endoscopic surgery skills of the physician. In Norway, all hospitals have at least one box trainer and some also have virtual reality simulators. This means that the physical tools for skills training are available. One of the advantages of simulators is immediate objective feedback on different performance parameters such as time, total path length, instrument movements and errors (bleeding and improper respect of tissue/tissue handling), and available learning curves.

We chose supracervical hysterectomy as the surgical procedure in the presented curriculum. The principle used in this study with a pre-set level of competence as a goal for the training, may be used for any surgical procedure. Departments that implement competence-based education for registrars, will after some time get experience with the average time needed for trainees to reach competence. This facilitates adding surgical training as a part of the registrars’ regular tasks. Consequently, the schedule of the trainees’ daily work can be organized accordingly. When a structured training curricula is implemented within a department, the trainees, the trainers and the employers will expect that the trainees practice, and that they have to reach a preset level of competence before they move on to surgical procedures in the operating room.

Another implication of introducing a curriculum with goal-oriented training, in contrast to time- based training, is the time spent on the simulator. When the trainees have reached the defined level of performance, they must move on to task of increased difficulty to further develop their skills (Strandbygaard et al., 2014; Guadagnoli and Lee, 2004). This knowledge should be used to make the working hours and surgical competence development of the residents as efficient as possible. When the registrars have reached the required competence level on the simulator, they move on to more advanced tasks or surgery, as spending time repeating procedures that are mastered on the simulator or on the box trainer have limited effect on further skills development.

The retention of skills is affected with time (Verdaasdonk et al., 2007; Guadagnoli and Lee, 2004). The retention of skills is a factor that must be taken into consideration for employers who choose to invest in training tools and implementation of a curriculum. It is essential to let the trainees move on to the operating room when they have finished the curriculum. In our opinion, this would furthermore improve the motivation of the trainees, as there is a clear and pre-defined goal of the training.

An education programme where you get a date for your first procedure when finishing the curriculum is an ideal situation. This might be a challenge to employers due to logistics. However, it might commit the trainees to implement the curriculum when they know the employer’s expectations and the positive consequences of the opportunity to do surgery.

The relative high variation of the duration of the training period in the study is related to individual variation of time to reach the proficiency level, but also working hours and logistics. We aimed for distributed training with sessions every week, since this is known to give the best results when developing psychomotor endoscopic skills (Van Dongen, 2011). The trainees performed their ordinary clinical duties on the ward during the training period. This led to an extension of training sessions for some of the participants.

The variation of the length of the training period might be a limitation of the study, but is also a strength because it increases the validity. It was performed in a busy clinical department without adjustment of clinical activity to facilitate training. In our opinion, this improves the quality and feasibility of this curriculum. Another strength of the study is that the surgical performance after training was assessed using validated assessment tools, and we could compare the performance with a comparable group of trainees that had not undergone systematic training.

Our results indicate that the accomplishment of a proficiency level on the simulator, predict that the trainees will perform surgery at a more automated level than trainees without training. Consequently, implementation of proficiency-based training in a dry lab before training in the operating room, might contribute to make our residents good enough for our patients (Campo et al., 2012). Use of validated scoring systems during surgery will further add value both to registrars and their supervisors in order to monitor surgical skills development and identify potential need for further training.

In conclusion, trainees who implemented the curriculum appeared to have a higher performance score on their first laparoscopic hysterectomy compared to trainees who did not perform structured training. By using the mean of the experienced surgeons’ performance, a proficiency level for any training procedure in order to enhance surgical skills and patient safety may be set.
